# Quantitative Fluorescence Imaging of Porphyrin Phospholipid Photobleaching and Light Activated Liposomal Doxorubicin Release Using Wide-field and Laparoscopic SFDI in an Ovarian Cancer Model

**DOI:** 10.21203/rs.3.rs-6030448/v1

**Published:** 2025-02-19

**Authors:** Rasel Ahmmed, Elias Kluiszo, Semra Aygun-Sunar, Matthew Willadsen, Hilliard L Kutscher, Jonathan F. Lovell, Ulas Sunar

**Affiliations:** 1 Department of Biomedical, Engineering, Stony Brook University, Stony Brook, NY 11794, USA; 2 POP Biotechnologies, Buffalo, NY 14228, USA; 3 Department of Biomedical Engineering, University at Buffalo, Buffalo, NY 14260, USA

**Keywords:** laparoscope, spatial frequency domain imaging, quantitative fluorescence imaging, doxorubicin drug concentration, light-triggered release, porphyrins, photodynamic therapy

## Abstract

Chemophototherapy (CPT) is an emerging cancer treatment that leverages the synergistic effects of photodynamic therapy (PDT) and chemotherapy. This approach utilizes photosensitizers like Porphyrin Phospholipid (PoP) and Doxorubicin (Dox) to enable phototriggered drug release and targeted tumor destruction. In this study, we present the development and validation of a wide-field laparoscopic spatial frequency domain imaging (SFDI) system, designed to improve intraoperative quantitative fluorescence imaging and monitoring of PoP photobleaching, a PDT-driven effect for tumor destruction, and light-activated Dox release, which facilitates targeted chemotherapeutic drug delivery in an ovarian cancer model. Compared to previous flexible endoscopic imaging methods, our laparoscopic SFDI system offers enhanced spatial coverage, enabling accurate wide-field optical property quantification in minimally invasive surgical settings. Using this system, we performed quantitative fluorescence imaging in vivo to obtain absolute concentrations of PoP and Dox fluorescence, correcting for tissue absorption and scattering effects. This capability allows for precise assessment of PoP photobleaching and Dox release kinetics with improved spatial resolution. Fluorescence imaging revealed a significant reduction in PoP concentration in tumor regions post-illumination, demonstrating the PDT-mediated photobleaching effect and successful light-triggered drug release activation for chemo-induced tumor destruction. The ability to differentiate PoP and Dox fluorescence in a laparoscopic system underscores its potential for real-time intraoperative monitoring of CPT efficacy. These findings establish wide-field laparoscopic SFDI as a promising tool for guiding minimally invasive photodynamic therapy and targeted drug delivery in clinical settings.

## 1. Introduction

Ovarian cancer remains one of the most lethal gynecological malignancies due to its high recurrence rates and peritoneal micrometastases, which are challenging to detect and treat effectively. Despite surgery and adjuvant chemotherapy, as many as 60% of patients can show occult disseminated ovarian disease [1–3]and systemic chemo can induce toxic side effects[4–7]. In addition, traditional imaging techniques, such as computed tomography (CT), magnetic resonance imaging (MRI), positron emission tomography (PET), and ultrasound, demonstrate less sensitive detection than reassessment surgeries[3, 8]. Thus, there exists an unmet need for both sensitive imaging technologies for improved detection and therapeutic approaches that selectively treat superficial tumors in the intraperitoneal cavity while avoiding healthy tissue.

Photodynamic therapy (PDT) has gained significant attention as a minimally invasive cancer treatment modality that leverages light, a photosensitizing agent, and molecular oxygen to generate cytotoxic reactive oxygen species (ROS) capable of selectively destroying tumor cells. Porphyrin-based compounds (photosensitizers-PS) are used for PDT and imaging because they strongly fluoresce, preferentially accumulate ~2–3 fold in malignant cells and have demonstrated some success in detecting subcutaneous tumors with qualitative imaging approaches [7, 9–13]. Porphyrin conjugate (Porphyrin-phospholipid-PoP) has been through over 10 human phototherapy clinical trials. Because superficial tumors can be on the epithelial surface and accessible via surgery, PDT is an attractive approach for fulfilling the adjunctive therapy role to surgery. However, high normal tissue toxicity has been a major obstacle for the clinical translation of PDT for ovarian cancer. Seminal works by Hasan [6, 7, 14–17] and Mordon [1, 18, 19] groups have shown epidermal growth factor receptor (EGFR) and folate-targeted approaches reducing the peritoneal tissue toxicity of PDT by using semi-quantitative imaging approaches in animal models.

To develop an image-guided system for the delivery and detection of PS, knowledge about PS concentrations at and near the target cells is essential. Our approach capitalizes on the high sensitivity and specificity of fluorescence contrast, the fact that Porphyrin are highly fluorescent [20], and the increased resolution of wide-field imaging. However, the accurate quantification of drug concentrations in tumors remains an ongoing challenge because the raw fluorescence signal is affected by tumor absorption and scattering properties, which confound the true fluorescence contrast [21, 22]. To correct this distortion, spectroscopic measurements can be taken to quantify tissue attenuation, but it is point-specific [23]. SFDI gathers tissue data over a wide area, allowing for accurate measurements in heterogeneous tissue like the intraperitoneal cavity.

SFDI can quantify both optical absorption and scattering during reflectance imaging mode [7]. Knowledge of the optical parameters can allow modeling the light dose distribution within the treatment field. In addition to light dosimetry, these parameters can provide the fluorescence correction factor for tissue attenuation so that one can extract absolute fluorescence concentration in vivo.

In this study, we employed a custom laparoscopic SFDI system to quantify the absolute concentration of PoP and Dox fluorescence concentrations in an ovarian cancer model. Doxorubicin (Dox), embedded in liposomal construct of PoP that enables spatially and temporally controlled release of Dox from liposomes using near infrared light [24]. The quantification of PoP fluorescence concentration revealed the significant photobleaching effect compared to the periphery. Next, we monitored the release of the Doxorubicin (Dox) and demonstrated significant release at the target sites compared to surrounding tissue.

## 2. Results

### 2.1. PoP Fluorescence Concentration Contrast and Photobleaching in Subcutaneous Tumors

**Figure 1.**
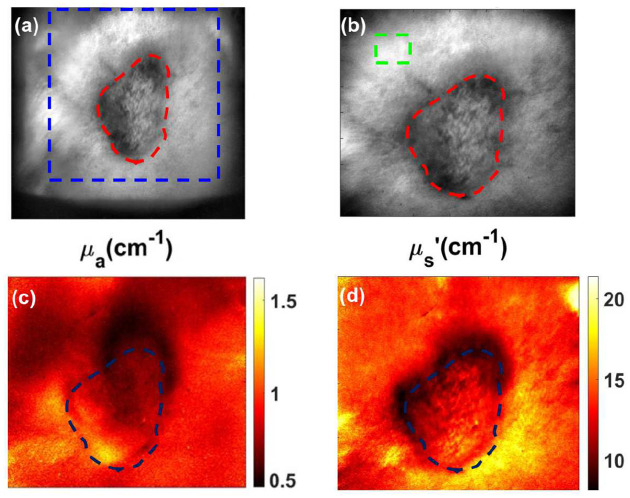
Representation of optical properties of the tumor and periphery (**a**) Raw image with specific region of interest (ROI) including both periphery and tumor region. (**b**) Diffuse reflectance image with the lesion and periphery marked. (**c** and **d**) Absorption and scattering map for 660 nm.

Figure 1(a) and (b) shows the diffuse reflectance image with the lesion and surrounding periphery area, while (c) and (d) shows the absorption and scattering maps at 660 nm, respectively. Fig. 1(c) showed higher absorption at the lesion compared to the surrounding periphery, while the scattering parameter at 660 nm was lower at the lesion in Fig. 1(d). Only one wavelength (660 nm) is presented for clarity; the 660 nm wavelength was chosen because this is the excitation wavelength of PoP used for PDT and Dox release.

**Figure 2.**
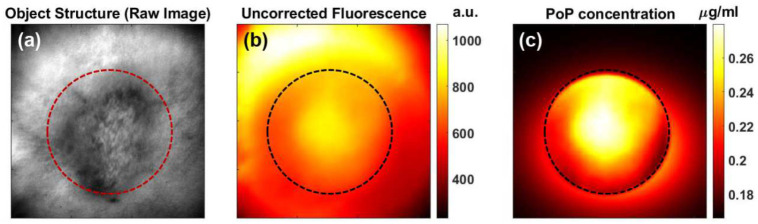
Representative images of a tumor after light administration to PoP (**a**) White light structural image showing the tumor area. (**b**) Uncorrected fluorescence image does not show localized contrast. (**c**) PoP fluorescence concentration indicating higher contrast between the tumor and surrounding area compared to the uncorrected fluorescence.

Figure 2 shows representative images of a tumor in a mouse 1h after administration of PoP. Figure 2(a) shows the whitelight image to indicate the tumor location. Tumors were large (~9 mm diameter). The uncorrected fluorescence shows unexpected tumor contrast (705.09±94.2a.u.-tumor vs 983.07± 58.51a.u.-periphery). However, the absolute PoP concentration image (after calibrating corrected fluorescence to absolute concentration) demonstrated a higher contrast in tumor, compared to peripheral tissue (0.24±0.026 μg/mL vs 0.183±0.0001 μg/mL, respectively).

**Figure 3.**
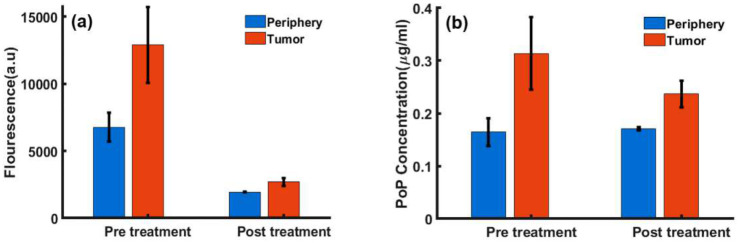
Extracted functional parameters from tumor and periphery before and after PDT (**a**) Bar plot showing the comparison of PoP fluorescence in the tumor vs peripheral tissue pre and post treatment light (**b**) Comparison of the PoP concentration (μg/ml) before and after treatment.

We also quantified the PS photobleaching in vivo. As shown in Figure 3(a), the PoP fluorescence amplitude was approximately 2.2 times higher in tumor tissue than in normal tissue before PDT. Following treatment light irradiation, fluorescence imaging revealed a significant (~75%) reduction in PoP concentration in the tumor region due to photobleaching [25], [26, 27], [28–30]. Figure 3b shows the absolute PoP concentration, indicating greater PoP uptake in tumor tissue compared to normal tissue, with a tumor-to-normal tissue uptake ratio of approximately 1.7, consistent with previously reported values [8]. Following PDT, the PoP concentration in the tumor decreased by about 25% due to photobleaching after treatment, while the drug concentration in normal tissue remained similar to the pre-PDT value.

### 2.2. Dox release in Mouse Carcass

#### 2.2.1. Dox imaging contrast

**Figure 4.**
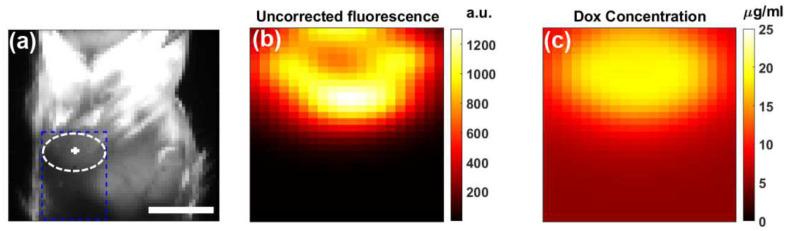
Representative Dox quantification in a mouse carcass. (**a**) White light image showing the structure of the PoP injection and release site. (**b**) Uncorrected Dox fluorescence image after 8-minute illumination. (**c**) Dox concentration image after 8-minute illumination

We then investigated Dox imaging contrast in a BALB/c mouse carcass. Figure 4(a) highlights the white light structural image, marking the PoP injection and Dox release sites. The injection site was exposed to treatment light for 8 minutes. The uncorrected fluorescence (Fig. 4(b)) and concentration (Fig. 4(c)) maps revealed differences in image contrast.

#### 2.2.2. Dox release kinetics

**Figure 5.**
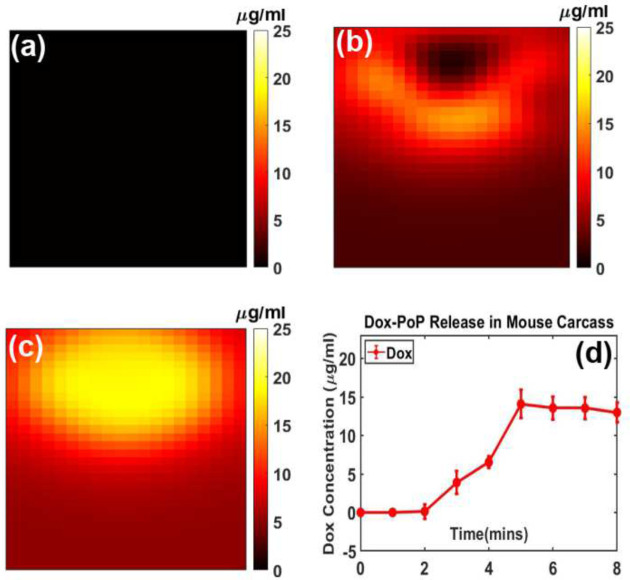
Dox releases kinetics in a mouse carcass. (**a**) Pre-treatment (**b**) 4 min post-treatment (**c**) 8 min post-treatment. (**d**) Complete Dox release kinetics curve with mean and standard deviation of the ROI.

We examined Dox release kinetics in a mouse carcass (Fig. 5). Figures 5(a), 5(b), and 5(c) display Dox concentrations at pre-treatment, 4 minutes post-treatment, and 8 minutes post-treatment, respectively, showing an increase from 6.53 ± 0.74 μg/mL to 13.01 ± 1.24 μg/mL distribution, likely due to variations in optical parameters, particularly scattering differences between the skin and intralipid. Additionally, auto fluorescence was subtracted from the total fluorescence signal to isolate the Dox contribution. Auto fluorescence accounted for approximately 60% of the initial background signal before treatment and about 10% of the peak fluorescence signal during treatment.

#### 2.2.3. Porphyrin photobleaching in Dox-PoP drug

**Figure 6.**
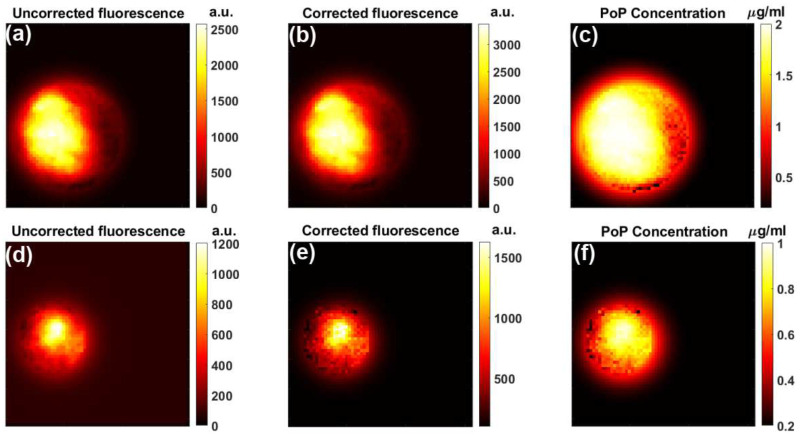
Porphyrin fluorescence pre and post-light treatment. (**a**-**c**) pre-treatment fluorescence and subsequent PoP concentration, (**d**-**f**) post-treatment fluorescence and subsequent PoP concentration.

We compared Porphyrin fluorescence before (Fig. 6a–c) and after (Fig. 6d–f) applying treatment light for Doxorubicin release, showing a decrease from 1.57 ± 0.37 μg/mL to 0.73± 0.14 μg/mL likely due to photobleaching from the strong treatment light at the excitation wavelength of the Porphyrin.

## 3. Discussion

In this study, wide-field and laparoscopic Spatial Frequency Domain Imaging (SFDI) measurements were performed to monitor the PoP and Dox fluorescence kinetics. While concurrent, real-time measurements during and shortly after PDT are highly desirable, they pose significant challenges. Previous studies have demonstrated that real-time measurements during PDT can provide predictive insights [8]. Non-contact methods, such as wide-field SFDI, enable real-time monitoring by “filtering out” treatment light during non-invasive measurements to extract relevant parameters.

However, achieving concurrent measurements during PDT remains complex. Unlike probe-based methods, which may interrupt treatment light and require fine adjustments during measurements, wide-field SFDI avoids physical interference with the treatment area. Furthermore, non-contact methods such as SFDI dual-channel endoscope systems [31] provide the flexibility to monitor tumors with flat surfaces, such as subcutaneous tumor models or skin, without physical disruption.

It is shown that a tumor has higher absorption and reasonable scattering coefficient which is minimally comparable for higher wavelength to peripheral tissue. Tumor absorption parameter was ~40% and scattering parameter was ~15% higher than surrounding normal tissue at 590 nm wavelength, which was approximately less than 10% for absorption and scattering at both excitation and emission wavelength of PoP.

Generally, skin layer covers tumor and consideration of skin layer may result in more accurate quantification of the tumor tissue. However, in this study, imaged tumors were larger, so skin layer effect is not minimal compared to human skin, though we did not consider this effect which might cause low accuracy.

In the laparoscopic SFDI data, there is a leveling off of Doxorubicin fluorescence occurring at 6 seconds of treatment time, likely due to completed activation of the PoP-liposomes. It is notable that the total activated Dox concentration shown in Fig. 5d did not reach the injected amount (equivalent to ~23 μg/mL of Dox). This is likely due to the shallow penetration depth of the higher frequency 490nm excitation light not probing the injection site completely. Limited penetration depth of 490nm light might also explain the heterogeneity of Doxorubicin signal in Fig. 5b as liposomes closer to the surface of the injection could be released first and then gradually mixed with the untreated volume over a longer period.

In Fig. 6, photobleaching in the subcutaneous administration of Dox-PoP resulted in a ~50% decrease in post-treatment, double the amount seen in intra-tumoral administration. This is likely due to the increased irradiation time applied to the subcutaneous injection, as well as differing PoP diffusion behavior in the intra-tumoral injection. Also of note is the difference in the injected vs. detected values of PoP prior to treatment light, with the intra-tumoral injection seeing 20% loss and the subcutaneous drug injection seeing 50% loss. This is potentially due to the different drug formulations and differing mouse strains used, with the BALB/c mouse in the drug analysis having thicker skin, and potential error from the hair removal process.

It should also be noted that all the reported values were obtained via post-processing with custom MATLAB software that fit the data to the model at each pixel. This process took between 5 and 10 minutes depending on the pixel binning and was performed outside the clinic. In total, the data acquisition time in the clinic was around 1 minute, pixel-by-pixel fitting for optical properties took 1–2 minutes per wavelength, and the multiwavelength fitting took 2–3 minutes. These times could be reduced by binning to fit fewer pixels. Utilizing a faster acquisition technique, such as single snapshot [32] or a faster processing technique such as the lookup table (LUT)[32] model recently proposed by Angelo et. al [33] could reduce the overall quantification time and provide results in the clinic. This near real-time feedback would be particularly useful for monitoring light-based therapies such as laser or photodynamic therapy.

## 4. Materials and Methods

### 4.1. Wide-field SFDI System

**Figure 7.**
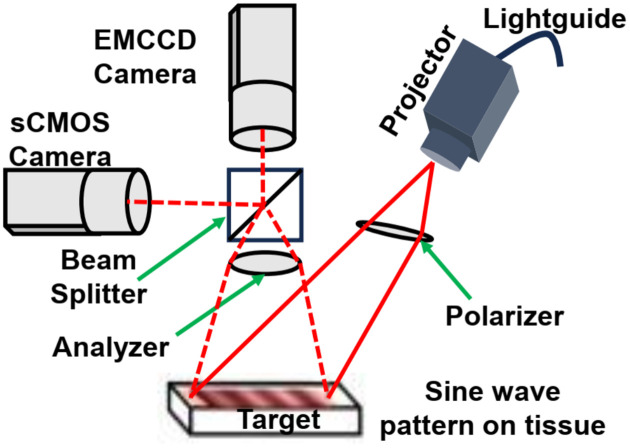
Schematic diagram of the imaging head showing the projector module, One charge-coupled device (CCD) and one sCMOS camera, beam splitter, polarizer, and analyzer. Light-emitting diode (LED) light is delivered with a light guide. Four LEDs are switched sequentially. Digital micromirror device generates sinusoidal patterns, patterns projected onto the skin surface by projector and reflected signal is detected by CCD cameras

A clinic-friendly SFDI system was constructed as shown in Figure 7. The system utilized four high-power, compact light-emitting diodes (LEDs) from the LCS series, each emitting light at 590 nm, 625 nm, 660 nm, and 740 nm (Mightex, Toronto, Ontario, Canada). A four-channel LED controller (Mightex) sequentially activated the selected excitation wavelength. The light was directed via a liquid light guide to a projector (Light Commander; Logic PD, Inc., Minneapolis, MN, USA) equipped with a digital micromirror device (DMD) module offering a resolution of 1024 × 768 pixels.

The DMD module generated sinusoidal patterns with three distinct phases (0, 2π/3, 4π/3) and 22 spatial frequencies ranging from 0 to 3.1764 cm^−1^. These patterns were projected onto the tumor surface, and the reflected light was captured by charge-coupled device (CCD) cameras. The cameras were aligned to focus on the same field of view as the projector, covering an area of 22 × 22 mm^2^. A rigid light shield with an imaging window was used to block ambient light and maintain a consistent distance from the target tissue. For imaging, the system incorporated two cameras positioned on either side of a 685 nm dichroic mirror (67–085; Edmund Optics, Barrington, NJ, USA). This design ensured precise imaging of the illuminated area. Fluorescence and reflectance imaging were performed simultaneously. The first sCMOS camera (Zyla Andor, Belfast, Ireland) captured reflectance images at 590, 630, and 660 nm, while a highly sensitive EMCCD camera (Luca; Andor, Belfast, Ireland) acquired reflectance images at 660nm and 740 nm and fluorescence data at 740 nm. Splitting the light at the 685 nm dichroic mirror allows for analysis of the projected excitation light by the sCMOS, and the fluorescent signal by the more sensitive EMCCD camera.

The cameras operated with an acquisition time of 100 ms per image, resulting in a total acquisition time of 27 seconds (calculated as 100 ms × 3 phases × 22 frequencies × 4 wavelengths). The system was fully automated using a custom LabView (National Instruments, Austin, TX, USA) software, which included subprograms to manage all system components. The software enabled automatic adjustments of LED intensities and exposure times for individual subjects.

To minimize specular reflections during reflectance imaging, cross-polarizers were placed in front of the projector and cameras. The LED light source intensity was maintained at <1 mW/cm^2^ to ensure safety.

The optical absorption and scattering properties were quantified by fitting an analytical, frequency-dependent diffuse reflectance model to the measured reflectance data across multiple spatial frequencies. This process utilized a reference phantom with known optical properties, as previously described [34]. All 22 spatial frequencies, ranging from 0 to 3.1764 cm^−1^, were included in the analysis. For each spatial frequency and wavelength, the three phase-shifted reflectance images were demodulated to isolate the spatially modulated component of the diffuse reflectance. The demodulated reflectance, which varies as a function of spatial modulation frequency, exhibits differing sensitivities to absorption and scattering parameters depending on the frequency. This allows SFDI to independently and accurately quantify both absorption and scattering. Using this approach, pixel-by-pixel fitting was performed to generate spatial maps of absorption and scattering properties.

### 4.2. Laparoscopic SFDI System

**Figure 8.**
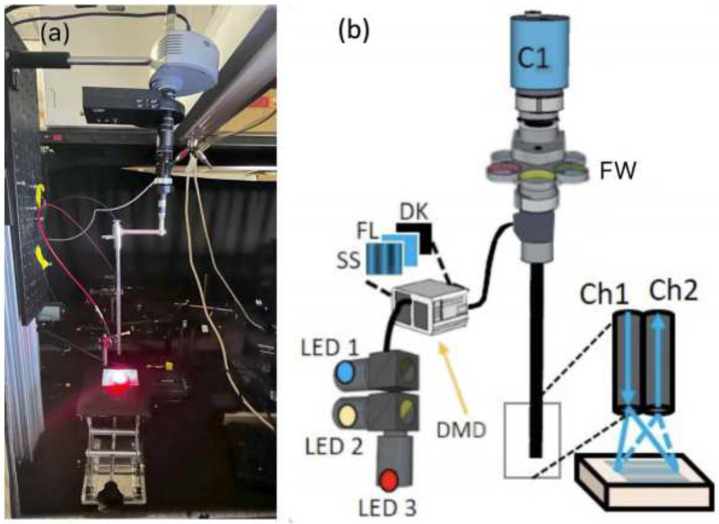
The experimental setup of Doxorubicin release in mouse carcass, (**a**) laparoscope projecting 490nm excitation light in blue and the 656nm treatment light in red, (**b**) laparoscope setup, showing LEDs and DMD with sinusoidal, fluorescent, and dark images projected, as well as the camera (C1), filter wheel placement (FW) and the projection (Ch1) and imaging (Ch2) channels.

The laparoscopic SFDI system was constructed as shown in Figure 8, with Figure 8a, showing mouse carcass imaging setup and Figure 8b detailing the projection and imaging components within the laparoscopic system.

Our laparoscopic fluorescence imaging and SFDI setup consisted of a modified DMD (LightCrafter 4500, Texas Instruments) to spatially modulate light to the appropriate sine wave patterns with three different phases (0, 2π /3, 4π/3) and five spatial frequencies from 0 to 2.5 cm^−1^ at a resolution of 1280× 800 pixels.

Light from three high-power LEDs at 490nm, 590nm, and 656nm was directed to the DMD through a liquid light guide (Mightex), with the LEDs and DMD being controlled remotely via Matlab. The 490 nm LED was used to excite Dox fluorescence and for acquiring SFDI optical properties at the Doxorubicin excitation peak, the 590nm LED was used to acquire optical properties at the Doxorubicin emission peak, and the 656nm light was used to excite Porphyrin fluorescence.

The DMD module generated sinusoidal patterns and projected them through a 2.4 mm imaging fiber with 13,000 elements (Asahi Kasei, Tokyo, Japan) which passed through the fixed laparoscope to the distal end facing the imaged surface. A custom objective lens at the tip of the fiber served to collimate light from the fiber and homogeneously project it out of the laparoscope and onto the tissue. Patterns from the DMD were reflected off the tissue and collected through the laparoscope optics, including the laparoscope itself (8912.43, R. Wolf), a zoom coupler (Accu-Beam, TTI Medical), two 30mm achromatic lenses, a filter wheel and an aperture (ThorLabs) before reaching the EMCCD camera (1004 ×1002 pixels, Luca, Andor, Belfast, Ireland). The camera was focused over the entire area of the projected SFDI pattern at a 3.2×3.2cm FOV. The optical design ensures an accurate sinusoidal projection for SFDI in a format using components currently used in laparoscopic surgery.

For acquiring SFDI data, no optical filters were used, with the LED output centered on the respective 490nm and 590nm excitation and emission wavelengths of Doxorubicin. When imaging Doxorubicin fluorescence, a 530nm longpass filter and a 593±40nm fluorescent filter were used to isolate Doxorubicin fluorescence from the excitation light. When imaging Porphyrin fluorescence, a 660±10 nm bandpass filter was applied to the 656nm LED to isolate PoP excitation signal, and a 716±40nm fluorescence filter was used to isolate PoP fluorescence. For each fluorescent measurement, a dark image with no projection light was taken to subtract noise during post-processing. Fluorescent images were taken with a 6 second exposure time at 16×16 camera binning, whereas SFDI measurements were taken at 2 seconds exposure per projection, at 8×8 camera binning. For SFDI measurements, two polarizing filters were applied to the tip of the endoscope, one for the projected patterns and the other for the reflected light, effectively cross polarizing to reduce spectral reflection. The polarizing filters were removed during fluorescence measurements. The optical absorption and scattering properties were extracted from SFDI images by fitting a frequency-dependent diffuse reflectance model to the measured reflectance data across multiple spatial frequencies as described prior, but with a total of 15 images at 5 frequencies ranging from 0 to 2.5 cm^−1^.

### 4.3. System Calibration

The wide-field SFDI instrument was tested on skin simulating phantoms with optical absorption (μ_a_) and scattering (μ_s’_) properties within the range of tissue at 660 nm, which is the common wavelength for PoP based PDT. The calibration phantoms used in this experiment were fabricated based on absorption and scattering parameters from prior work [35]. Bulk optical parameters were quantified by fitting frequency-dependent reflectance data with modified frequency-domain diffusion models by using a reference phantom with known optical properties [34].

The laparoscopic SFDI system was characterized with multiple calibration phantoms made by titrating the optical properties ranging from μ_a_= 0.5cm^−1^ to 1.5cm^−1^ and μ_s’_= 10cm^−1^ to 30cm^−1^ at 490nm and 590nm. The mean percent error in quantifying the absorption parameter was always less than 10%. Fluorescence phantoms were prepared by titrating the Dox concentration, with a 0.5mg/mL free-Dox solution used to acquire fluorescence values at 2, 4, 6, 8 μg/mL in phantoms of varied optical properties. Reconstructed raw fluorescence values with respect to concentration were used as a calibration curve to obtain absolute Dox concentrations as previously described [36].

### 4.4. Subcutaneous tumor culture in mouse model

8 female nude mice were inoculated subcutaneously with 10^7^ SKOV-3 cells. After reaching approximately 9 mm in diameter, the tumors were injected with 0.4μg/ml PoP in 400μL 5% dextrose solution. For imaging, mice were placed on a heating pad and anesthetized with Isoflurane. The injection was left to diffuse within the tumor for 1 hour before fluorescence imaging with 660nm LED through the 740nm EMCCD filter. A treatment light at 660nm was applied to the injection site for 5 minutes, after which fluorescence images were taken again using the same parameters.

### 4.5. Preparation of long-circulating Dox in PoP Liposomes

The details of our PoP Dox liposome formulation have been described in [24, 27, 37]. PoP-liposomes incorporated PoP photosensitizer in a recent study for the development of improved peptide-based cancer vaccines, underscoring the versatility of the drug delivery platform [26]. Briefly, PoP-liposomes were synthesized from pyro-lipid through esterification of pyro with lyso-C16-PC, using 1-Ethyl-3-(3-dimethylaminopropyl)carbodiimide (EDC) and 4-dimethylaminopyridine (DMAP) in chloroform. The liposomes were formed by dispersing Porphyrin-lipid, PEGylated-lipid, cholesterol, and distearoylphosphatidylcholine in chloroform, followed by solvent evaporation. A 20 mg/mL lipid solution was extruded through a high-pressure lipid extruder with a 250 mM ammonium sulfate solution using polycarbonate membranes of 0.2, 0.1, and 0.08 μm pore size, sequentially stacked and passed through the extruder 10 times. Free ammonium sulfate was removed by overnight dialysis in a 10% sucrose solution with 10 mM HEPES at pH 7. Dox was loaded by incubating the liposomes at 60°C for 1 hour, achieving a loading efficacy of over 90% as confirmed by G-75 column tests. The self-assembly status and elution position of PoP-liposomes were tracked using 420 nm excitation and 670 nm emission, while Dox was detected using 480 nm excitation and 590 nm emission in a fluorescence plate reader (TECAN Safire).

### 4.6. Dox release quantification and PoP photo bleaching by laparoscopic SFDI in mouse model

A recently sacrificed BALB/c mouse was acquired for simulated in vivo measurements. The mouse was placed on an imaging platform with the shaved injection site centered within the 3.2×3.2cm FOV of the laparoscope. SFDI measurements at the 490nm excitation and 590nm emission wavelengths of Doxorubicin were performed prior to injection. The mouse was injected subcutaneously with 50 μL of a lightly scattering intralipid medium (s’ = 5cm^−1^ at 490nm) containing 23.1μg/mL PoP-Liposomes, and SFDI measurements were performed again post injection. A treatment light at 657nm at a fluence rate of 330mW/cm^2^ was directed onto a 1cm area centered on the injection site, and fluorescence measurements were acquired every 1 minute to assess drug release dynamics. PoP fluorescence images were acquired immediately prior to and after applying treatment light to assess photo bleaching.

### 4.7. Attenuation-Compensated Fluorescence Analysis

By using SFDI in fluorescence imaging mode, photosensitizer (PS) fluorescence can allow quantification of PS concentration by accurately compensating for light attenuation at both excitation and emission wavelengths. The quantification of PoP fluorescence concentration was performed using the Gardner model [38], which corrects the raw fluorescence signal by accounting for optical absorption (μ_a_) and scattering (μ_s’_) losses at both excitation and emission wavelengths. In this model, the fluorescence correction factor, X_1D_(ex,em), is determined using the quantified optical parameters from SFDI measurements, where X_1D_(ex,em), represents the effective path length during the penetration of excitation light and the escape of emitted fluorescence from the tissue. The corrected fluorescence was then calculated as F_corr_= F_raw_/X_1D_, where F_raw_ is the measured raw signal, X_1D_ is the correction factor, and F_corr_ is the fluorescence corrected for optical absorption, scattering, and light propagation. Using the calibration factor, the corrected fluorescence was translated into PoP concentrations. Fluorescence imaging can also be used for monitoring PDT response because PS fluorescence changes during PDT, and these changes may be indicative of PDT response.

To quantitatively compare tumor regions with surrounding normal tissue, image analysis was performed using a hand-drawing tool function (*imfreehand/impoly*) in MATLAB (MathWorks, Inc., Natick, MA, USA). Regions of interest (ROIs) for both tumor and peripheral tissue were selected based on reflectance maps at 590, 625, 660, 740 nm. Statistical metrics, including mean and standard deviation, for each ROI are summarized in a barplot. The analysis revealed contrasts between tumor and peripheral tissue, with tumor ROIs exhibiting higher mean absorption parameters and lower mean scattering. laparoscopic SFDI data was processed similarly, with ROI’s pertinent to the injected drug area being selected for analysis at each fluorescence acquisition. The mean value of this area for each minute was used to determine Dox release kinetics, and images taken at the PoP fluorescence wavelength before and after were compared to asses Porphyrin photo bleaching. Data is summarized with Dox release leveling off after sufficient light dosage, and Porphyrin fluorescence decreasing due to photo bleaching by drug activation light.

## 5. Conclusions

This preliminary study demonstrates the feasibility and effectiveness of using SFDI for monitoring photo bleaching effects during PDT in subcutaneous tumor models. By quantifying the spatial distribution of PoP concentration pre- and post-treatment, the preliminary findings reveal the selective activation of PoP in the tumor region and its subsequent photo bleaching. The high intra-tumoral optical absorption underscores the importance of tumor optical properties in influencing therapeutic response. The ability of SFDI to provide non-invasive, spatially resolved insights into PDT dynamics highlights its potential as a critical tool for enhancing treatment precision, reducing collateral damage, and improving patient outcomes. Use in a laparoscopic SFDI system with light-activated therapeutics shows feasibility for controlled cancer treatment during surgery, with combined quantification of doxorubicin/Porphyrin fluorescence allowing for more accurate analysis of drug perfusion and efficacy. Future studies should investigate its application in clinical settings and explore its integration with other imaging modalities for comprehensive tumor characterization.

## Data Availability

The data that support the findings of this study are available from the corresponding author upon reasonable request.

## References

[1] Azais H, S. C., Tardivel M, Kerdraon O, Stallivieri A, Frochot C, Betrouni N, Collinet P, Mordon S., Assessment of the specificity of a new folate-targeted photosensitizer for peritoneal metastasis of epithelial ovarian cancer to enable intraperitoneal photodynamic therapy. A preclinical study. Photodiagnosis Photodyn Ther, 2016. 13: p. 130–138. 26200606 10.1016/j.pdpdt.2015.07.005

[2] Longmire M, K. N., Ogawa M, Choyke PL, Kobayashi H., Multicolor in vivo targeted imaging to guide real-time surgery of HER2-positive micrometastases in a two-tumor coincident model of ovarian cancer. Cancer Sci, 2009. 100(6): p. 1099–1104. 19302283 10.1111/j.1349-7006.2009.01133.x PMC2736472

[3] Sugiyama T, N. T., Komai K, Nishimura H, Yakushiji M, Nishimura H., Comparison of CA 125 assays with abdominopelvic computed tomography and transvaginal ultrasound in monitoring of ovarian cancer. Int J Gynecol Obstet 1996. 54(3): p. 251–256. 10.1016/0020-7292(96)02721-x 8889633

[4] Chan JK, M. B., Cuccia D, Pham H, Kimel S, Gu M, Hammer-Wilson MJ, Liaw LHL, Osann K, DiSaia PJ, Berns M, Tromberg B, Tadir Y., Laparoscopic photodynamic diagnosis of ovarian cancer using 5-aminolevulinic acid in a rat model. Gynecol Oncol 2002. 87(1): p. 64–70. 12468344 10.1006/gyno.2002.6793

[5] Luo D, C. K., Miranda D, Lovell JF., Chemophototherapy: An Emerging Treatment Option for Solid Tumors. Adv Sci., 2017. 4(1). 10.1002/advs.201600106 PMC5238751 28105389

[6] Rizvi I, C. J., Evans CL, Abu-Yousif AO, Muzikansky A, Pogue BW, Finkelstein D, Hasan T., Synergistic enhancement of carboplatin efficacy with photodynamic therapy in a three-dimensional model for micrometastatic ovarian cancer. Cancer Research, 2010. 70(22): p. 9319–9328. 21062986 10.1158/0008-5472.CAN-10-1783 PMC3057933

[7] Van Dam GM, T. G., Crane LMA, Harlaar NJ, Pleijhuis RG, Kelder W, Sarantopoulos A, De Jong JS, Arts HJG, Van Der Zee AGJ, Bart J, Low PS, Ntziachristos V., Intraoperative tumor-specific fluorescence imaging in ovarian cancer by folate receptor-α targeting: First in-human results. Nat Med, 2011. 17(10): p. 1315–1319. 21926976 10.1038/nm.2472

[8] Rose PG, F. P., Miraldi F, Abdul-Karim FW., Positive emission tomography for evaluating a complete clinical response in patients with ovarian or peritoneal carcinoma: Correlation with second-look laparotomy. Gynecol Oncol 2001. 82(1): p. 17–21. 11426956 10.1006/gyno.2001.6246

[9] Boussios S, P. G., Katsanos K, Pavlidis N., Systemic treatment-induced gastrointestinal toxicity: Incidence, clinical presentation and management. Ann. Gastroenterol., 2012. 25(2): p. 106–118. 24713845 PMC3959393

[10] Mo W, R. D., Sunar U., Imaging a photodynamic therapy photosensitizer in vivo with a time-gated fluorescence tomography system. J Biomed Opt., 2012. 17(7). 10.1117/1.JBO.17.7.071306 PMC3381019 22894467

[11] Ozturk MS, R. D., Sunar U, Intes X., Mesoscopic Fluorescence Tomography of a Photosensitizer (HPPH) 3D Biodistribution in Skin Cancer. Acad Radiol., 2014. 21(2): p. 271–280. 24439340 10.1016/j.acra.2013.11.009

[12] Staropoli N, C. D., Botta C, Fiorillo L, Grimaldi A, Lama S, Caraglia M, Salvino A, Tassone P, Tagliaferri P., Pegylated liposomal doxorubicin in the management of ovarian cancer: A systematic review and metaanalysis of randomized trials. Cancer Biol Ther 2014. 15(6): p. 707–720. 24658024 10.4161/cbt.28557 PMC4049787

[13] Sunar U, R. D., Morgan J, Zeitouni N, Henderson BW., Quantification of PpIX concentration in basal cell carcinoma and squamous cell carcinoma models using spatial frequency domain imaging. Biomed Opt Express, 2013. 4(4). 10.1364/BOE.4.000531 PMC3617715 23577288

[14] Celli JP, R. I., Blanden AR, Massodi I, Glidden MD, Pogue BW, Hasan T., An imaging-based platform for high-content, quantitative evaluation of therapeutic response in 3D tumour models. Sci Rep 2014. 4. 10.1038/srep03751 PMC3894557 24435043

[15] Spring BQ, B. S.R., Zheng LZ, Mai Z, Watanabe R, Sherwood ME, Schoenfeld DA, Pogue BW, Pereira SP, Villa E, Hasan T., A photoactivable multi-inhibitor nanoliposome for tumour control and simultaneous inhibition of treatment escape pathways. Nat Nanotechnol 2016. 11(4): p. 378–387. 26780659 10.1038/nnano.2015.311 PMC4821671

[16] Wang S, H. G., Scholzen T, Zhang Z, Vogel A, Hasan T, Rahmanzadeh R., A light-controlled switch after dual targeting of proliferating tumor cells via the membrane receptor EGFR and the nuclear protein Ki-67. Sci Rep 2016. **6**. 10.1038/srep27032 PMC4887907 27246531

[17] Zhong W, C. J., Rizvi I, Mai Z, Spring BQ, Yun SH, Hasan T., In vivo high-resolution fluorescence microendoscopy for ovarian cancer detection and treatment monitoring. Br J Cancer 2009. 101(12): p. 2015–2022. 19920823 10.1038/sj.bjc.6605436 PMC2795438

[18] Azais H, Q. G., Bonner C, Kerdraon O, Tardivel M, Jetpisbayeva G, Frochot C, Betrouni N, Collinet P, Mordon S., Fischer 344 Rat: A Preclinical Model for Epithelial Ovarian Cancer Folate-Targeted Therapy. Int J Gynecol Cancer 2015. 25(7): p. 1194–1200. 26244757 10.1097/IGC.0000000000000497

[19] Guyon L, A. M., Collinet P, Mordon S., Photodiagnosis and photodynamic therapy of peritoneal metastasis of ovarian cancer. Photodiagnosis Photodyn Ther 2012. 9(1): p. 16–31. 22369725 10.1016/j.pdpdt.2011.08.003

[20] Bogaards A, S. H., Trachtenberg J, Wilson BC, Lilge L., In vivo quantification of fluorescent molecular markers in real-time by ratio imaging for diagnostic screening and image-guided surgery. Lasers Surg Med 2007. 39(7): p. 605–613. 17868102 10.1002/lsm.20525

[21] Yang B, S. M., Tunnell JW., Attenuation-corrected fluorescence extraction for image-guided surgery in spatial frequency domain. J Biomed Opt 2013. 18(8). 10.1117/1.JBO.18.8.080503 PMC3745168 23955392

[22] Yang VX, M. P., Herman P, Wilson BC., A multispectral fluorescence imaging system: design and initial clinical tests in intraoperative Photofrin-photodynamic therapy of brain tumors. Lasers Surg Med 2003. 32(3): p. 224–232. 12605430 10.1002/lsm.10131

[23] Baran T.M.F., T. H., Recovery of intrinsic fluorescence from single-point interstitial measurements for quantification of doxorubicin concentration. Lasers Surg. Med., 2013. 45: p. 542–550. 24037853 10.1002/lsm.22166 PMC3936788

[24] Luo D., C. K.A., Razi A., Geng J., Shao S., Giraldo D., Sunar U., Ortega J., and Lovell J. F., Doxorubicin encapsulated in stealth liposomes conferred with light-triggered drug release. Biomaterials, 2016. 75: p. 193–202. 26513413 10.1016/j.biomaterials.2015.10.027 PMC4644481

[25] Bellnier DA, H. B., Pandey RK, Potter WR, Dougherty TJ., Murine pharmacokinetics and antitumor efficacy of the photodynamic sensitizer 2-[1-hexyloxyethyl]-2-devinyl pyropheophorbide-a. J Photochem Photobiol B., 1993. 20(1): p. 55–61. 8229470 10.1016/1011-1344(93)80131-r

[26] He X., Z. S., Huang W. C., Seffouh A., Mabrouk M. T., Morgan M. T., Ortega J., Abrams S. I., and Lovell J. F., A Potent Cancer Vaccine Adjuvant System for Particleization of Short, Synthetic CD8+ T Cell Epitopes. ACS Nano 2021. 15(3): p. 4357–4371 33606514 10.1021/acsnano.0c07680 PMC10184788

[27] Sunar U., K. J., Rohrbach D. J., Carter K. A., Luo D., Shao S., Lele S., and Lovell J. F, Light-triggered doxorubicin release quantified by spatial frequency domain imaging and diffuse optical spectroscopy, in Optics InfoBase Conference Papers. 2016.

[28] Georgakoudi I, N. M., Foster TH., The mechanism of Photofrin photobleaching and its consequences for photodynamic dosimetry. Photochem Photobiol., 1997. 65(1): p. 135–144. 9066293 10.1111/j.1751-1097.1997.tb01889.x

[29] Sheng C, H. P., Hasan T, Pogue BW., Photobleaching-based dosimetry predicts deposited dose in ALA-PpIX PDT of rodent esophagus. Photochem Photobiol., 2007. 83(3): p. 738–748. 17576383 10.1562/2006-09-07-RA-1033

[30] Wilson BC, P. M., Lilge L., Implicit and explicit dosimetry in photodynamic therapy:a new paradigm. Lasers Med Sci., 1997. 12(3): p. 182–199. 20803326 10.1007/BF02765099

[31] Kress J, R. D., Carter KA, Luo D, Poon C, Aygun-Sunar S, Shao S, Lele S, Lovell JF, Sunar U., A dual-channel endoscope for quantitative imaging, monitoring, and triggering of doxorubicin release from liposomes in living mice. Sci Rep., 2017. 7(1). 10.1038/s41598-017-15790-y PMC5686102 29138489

[32] van de Giessen M., A. J.P., and Gioux S., Real-time, profile-corrected single snapshot imaging of optical properties. Biomed. Opt. Express 2015. 6(10): p. 4051–4062. 26504653 10.1364/BOE.6.004051 PMC4605062

[33] Angelo J., V. C.R., Lee B. T., Bigio I. J., and Gioux S., Ultrafast optical property map generation using lookup tables. J. Biomed. Opt., 2016. 21(11). 10.1117/1.JBO.21.11.110501 PMC5997006 27901550

[34] Cuccia DJ, B. F., Durkin AJ, Ayers FR, Tromberg BJ., Quantitation and mapping of tissue optical properties using modulated imaging. J Biomed Opt 2009. 14(2). 10.1117/1.3088140 PMC2868524 19405742

[35] Rohrbach DJ, M. D., Huihui J, Saager R, Keymel K, Paquette A, Morgan J, Zeitouni N, Sunar U., Preoperative mapping of nonmelanoma skin cancer using spatial frequency domain and ultrasound imaging. Acad Radiol., 2014. 21(2). 10.1016/j.acra.2013.11.013 PMC3960981 24439339

[36] Kress J., R. D.J., Carter K. A., Luo D., Shao S., Lele S., Lovell J. F., and Sunar U., Quantitative imaging of light-triggered doxorubicin release. Biomed. Opt. Express, 2015. 6(9). 10.1364/BOE.6.003546 PMC4574678 26417522

[37] Gordon A.N.e.a., Recurrent epithelial ovarian carcinoma: a randomized phase III study of pegylated liposomal doxorubicin versus topotecan. J Clin Oncol 2001. 19: p. 3312–3322 11454878 10.1200/JCO.2001.19.14.3312

[38] Gardner CM, J. S., Welch AJ., Fluorescence spectroscopy of tissue: recovery of intrinsic fluorescence from measured fluorescence. Appl Opt 1996. 35(10). 10.1364/AO.35.001780 21085302

